# 

**Published:** 1886-09

**Authors:** 


					﻿SEPTEMBER, 1886-
_
OLD SERIES
—Vol.	__
'V
<Z 1855 O'*
NEW SERIES
k Vol. III.—No. 7.

fcCASOlfflk
PJPwWmiwM
i^»'’aO\ ^~a« E II IK v 11 Iv>'•Ari' \>
/*“*s JT	Jr	X -.	\JMk \ ^JBM
■’h r //	1 • i1 - t» .Jr	- " ’* '
—Il ^3ri*AtfV9’rf
<P W.F.WESTMOREtANaW> /
A1A H.VM.MlLLER.M.D.LL.D.JKu
Brp	James a.graymjj.’W
W <5--------------
MjBLISHED By JAMES P.HARRlSON&COg
W_________________Atlanta, ga. JM
SUBSCRIPTION: S2.SO A YEAR.
				

